# ZNF521 Is Correlated with Tumor Immune Cell Infiltration and Act as a Valuable Prognostic Biomarker in Gastric Cancer

**DOI:** 10.1155/2022/5288075

**Published:** 2022-10-19

**Authors:** Li Li, Zheng-Hui Liu, Hui-Ju Wang, Lei Wang, Guo-Qing Ru, Yuan-Yu Wang

**Affiliations:** ^1^General Surgery, Cancer Center, Department of Gastrointestinal and Pancreatic Surgery, Zhejiang Provincial People's Hospital (Affiliated People's Hospital, Hangzhou Medical College), Zhejiang, Hangzhou 310014, China; ^2^Key Laboratory of Gastroenterology of Zhejiang Province, Zhejiang Provincial People's Hospital, Affiliated People's Hospital, Hangzhou Medical College, Hangzhou, Zhejiang, China; ^3^Graduate School of Bengbu Medical University, Bengbu 233030, China; ^4^Department of Gastrointestinal Surgery, Central Hospital Affiliated to Shandong First Medical University, Jinan 250013, China; ^5^Cancer Center, Department of Pathology, Zhejiang Provincial People's Hospital (Affiliated People's Hospital, Hangzhou Medical College), Zhejiang, Hangzhou 310014, China

## Abstract

**Aim:**

To explore the correlations between the expression of zinc finger protein 521 (ZNF521) with immune invasion and prognosis of gastric cancer.

**Methods:**

Expression of ZNF521 was examined by immunohistochemistry in gastric cancer cases. Kaplan–Meier plotter was used to determine the relationships between ZNF521 and prognosis. TIMER and GEPIA were used to analyze the correlation between ZNF521 expression and gene markers of immune cell infiltration.

**Results:**

The expression of ZNF521 was up-regulated in gastric cancer samples. Kaplan–Meier analysis indicated that higher expression of ZNF521 was associated with poor prognosis. The expression of ZNF521 was correlated with infiltrating levels of CD4+ T and CD8+ T cells, macrophages, neutrophils, and dendritic cells in gastric cancer, which also correlated with diverse immune marker sets.

**Conclusions:**

ZNF521 is correlated significantly with immune cell infiltration and is a valuable biomarker for prognosis in gastric cancer.

## 1. Introduction

The incidence and mortality rate of gastric cancer ranked 5th and 3rd worldwide, respectively, in 2018, and their respective rates in China were 44.1% and 49.9% [1]. In recent years, studies have shown that cancer is closely related to autoimmunity, and immunotherapy is a new treatment method that has attracted extensive attention in the field of cancer treatment [2–5]. While immune infiltration in the tumor microenvironment is the basis of immunotherapy and plays a key role in tumorigenesis and development, it also affects patient prognosis [6]. Immune checkpoint inhibitors that target Programmed Cell Death Protein 1 (PD-1) or Programmed Cell Death Ligand 1 have greatly improved outcomes for patients with many types of cancer; however, only 20–40% of patients benefit from these therapies [7]. Therefore, it is necessary to improve the efficacy of immunotherapy and to find indicators of immune infiltration and explore their underlying mechanisms of activity.

Zinc finger protein 521 (ZNF521) encodes a transcription factor with a zinc finger domain that is widely expressed in many tissues and plays important roles in tumor formation and development [8, 9]. ZNF521 has been identified as a potent inhibitor of B-cell factor 1 (EBF1) and has emerged as a factor potentially associated with the development of B-cell leukemia [10]. Previous studies have found that ZNF521, which is downregulated by miR-802, suppresses malignant progression of hepatocellular carcinoma by regulating Runx2 expression [11]. ZNF521 can also arrest apoptosis and enhance the proliferation, migration, and invasion of gastric cancer cells via regulating microRNA-204-5p [12].

In this study, we detected ZNF521 expression in gastric cancer by immunohistochemistry (IHC) and analyzed the correlation between its expression with pathological parameters and prognosis. Kaplan–Meier plotter databases were used to analyze the expression of ZNF521 and its correlation with the prognosis of gastric cancer. Moreover, we analyzed the correlation between ZNF521 expression and infiltrating immune cells in different tumor microenvironments through the tumor immune database TIMER. ZNF521 promotes the malignant characteristics of gastric cancer cells in part by interacting with immune infiltrating cells. The findings of this report elucidate the important role of ZNF521 in stomach adenocarcinoma (STAD) and suggest a potential relationship and mechanistic link between ZNF521 and tumor immune interaction. We focused on ZNF521 expression and its relationship with clinicopathological features and prognosis to reveal the relationship between ZNF521 and tumor immune infiltration.

## 2. Materials and Methods

### 2.1. Immunohistochemistry

Tissue microarrays were performed as described in our previous study [13]. This study was approved and monitored by the ethics committee of Zhejiang Provincial People's Hospital. Streptavidin-peroxidase and high pressure immunohistochemical methods were adopted to examine antibody expression. All formalin-fixed, paraffin-embedded tissue sections were deparaffinized in an oven at 60°C overnight, and then further dewaxed in xylene. The pressure cooker antigen repairing method in citrate buffer solution was performed, and then 3% hydrogen peroxide was used to inhibit endogenous peroxidases. Sections were incubated with mouse anti-ZNF521 (1 : 1,000; Novus Biologicals, Littleton, CO, USA) overnight at 4°C.

### 2.2. Kaplan–Meier Plotter Database

The Kaplan–Meier Plotter can assess the effect of 54,000 genes (mRNAs, miRNAs, and protein-coding) on survival in 21 cancer types including breast (*n* = 7,830), ovarian (*n* = 2,190), lung (*n* = 3,452), and gastric (*n* = 1,440) cancers. Sources for the databases included the Gene Expression Omnibus, European Genome-Phenome Archive, and The Cancer Genome Atlas (TCGA). The primary purpose of the tool is meta-analysis-based discovery and validation of survival biomarkers. We used this database to assess the relationship between TNF521 expression and patient outcomes (http://kmplot.com/analysis/) [14, 15]. The log-rank test was used for statistical analyses, with *P* < 0.05 was considered significant differences.

### 2.3. TIMER Database

TIMER (https://cistrome.shinyapps.io/timer/) is a database designed for analyzing immune cell infiltrates in multiple cancer types [16]. It includes 10,897 samples across 32 different cancer types from TCGA. The correlation between ZNF521expression levels and immune infiltrates including B cells, CD4+ T cells, CD8+ T cells, neutrophils, macrophages, and dendritic cells (DCs) were explored using the gene module in various cancer types [17, 18]. The correlation between genetic markers of tumor-infiltrating immune cells and ZNF521 expression was analyzed using the correlation module [19–22]. ZNF521expression was plotted on the *x*-axis, and the expression of related marker genes were represented as gene symbols on the *y*-axis. Correlation coefficients were estimated using Spearman's correlation test. Gene expression levels were shown as log2 RNA-seq by Expectation-Maximization.

### 2.4. GEPIA Database

GEPIA is an online database that facilitates the standardized analysis of RNA-seq data from 9,736 tumor samples and 8,587 normal control samples from TCGA and Genotype-Tissue Expression data sets (http://gepia.cancer-pku.cn/index.html) [23]. The RNA-seq data of all 9,736 tumor samples and 8,587 normal control samples were analyzed. Therefore, we used the database to assess the association between ZNF521 expression and prognosis in a variety of tumor types and to further assess the association between ZNF521 expression and specific markers related to tumor immune cell infiltration.

### 2.5. Statistical Analysis

Prognoscan-, Kaplan–Meier-, and GEPIA-generated survival curves are displayed with hazard ratios (HRs) with 95% confidence intervals (CIs) and *P*- or Cox *P*-values from log-rank tests. The results generated in Oncomine are displayed as *P*-values, fold changes, and levels. Correlations of gene expression were evaluated by Spearman's correlation, with statistical significance and the correlation strength being determined by the following absolute value guidelines: 0.00–0.19, very weak; 0.20–0.39, weak; 0.40–0.59, medium; 0.60–0.79, strong; and 0.80–1.0, very strong. Statistical analyses were performed using SPSS 19.0 software (SPSS Inc., Chicago, IL, USA). Correlations between ZNF521 expression and clinicopathological parameters were analyzed by the Chi-square test or *t*-test. Survival curves were estimated by the Kaplan–Meier method and compared using the log-rank test. Statistical significance was set at *P* < 0.05.

## 3. Results

### 3.1. ZNF521 Expression in Gastric Cancer and Normal Tissues

**Figure 1:**
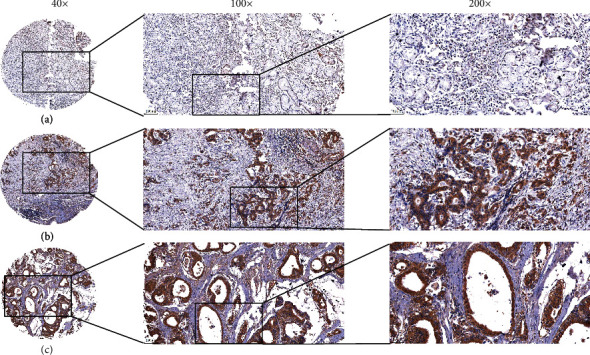
The expression level of ZNF521 in gastric cancer and noncancerous tissues by IHC. (a) ZNF521 was mainly localized in the cytoplasm of cancer cells, weakly expressed in noncancerous tissues, magnification ×200. (b)–(d) ZNF521 was highly expressed in moderately differentiated adenocarcinoma and poorly differentiated adenocarcinoma, magnification ×200.

We analyzed ZNF521 expression in normal and tumor tissues by IHC. ZNF521 expression levels in gastric cancer tissues were 58.7% (256/436), which was higher than in normal tissues (20.6% [19/92]). ZNF521 was primarily localized within the cytoplasm of cancer cells, and ZNF521 expression in non-tumor mucosa was also recorded (Figure 1). According to the IHC scores, ZNF521 expression was higher in gastric cancer tissues than in non-tumor gastric tissues. There was also a significant difference between the gastric cancer group and non-tumor mucosa (*P* < 0.05).

### 3.2. Relationship between ZNF521 Expression and Clinicopathological Parameters

To explore the clinical significance of ZNF521 in gastric cancer and to analyze correlations between ZNF521 expression and the clinical characteristics of gastric cancer patients, patients were divided into two groups according to their ZNF521 expression levels: the high and low ZNF521 expression groups.

ZNF521 was highly expressed in 58.7% of patients, and its expression was significantly correlated with tumor location, tumor size, Lauren classification, histological classification, histological differentiation, depth of invasion, regional lymph nodes, distant metastasis, and tumor node metastasis (TNM) stage (*P* < 0.01; Table 1). ZNF521 expression was not significantly correlated with age or sex (*P* > 0.05; Table 1).

### 3.3. Correlation between ZNF521 Expression and Prognosis

**Figure 2:**
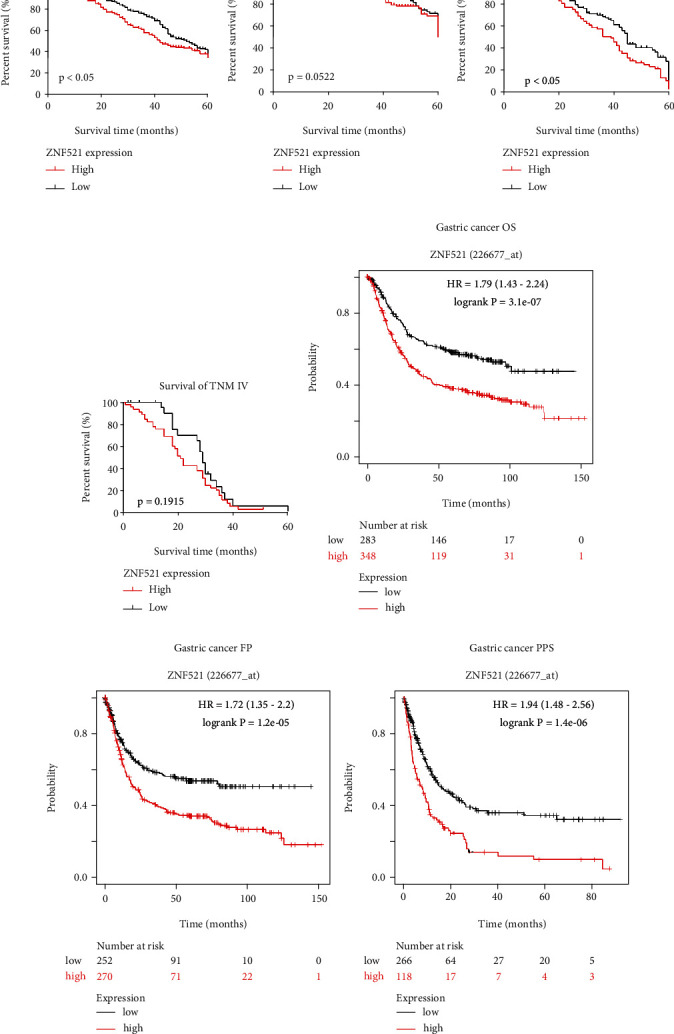
The correlation between ZNF521 expression and patient prognosis. (a)–(d) Kaplan–Meier curves with univariate analyses (log-rank) for the patients with low ZNF521 expression versus the high ZNF521 expression tumors. (e)–(g) The correlation between ZNF521 and prognosis of gastric cancer in the Kaplan–Meier plotter databases (K–M) survival curves of OS, FP, and PPS in Gastric cancer.

The Kaplan–Meier method was used to analyze the effects of ZNF521 on the prognosis of gastric cancer patients. The results indicated that patients with high ZNF521 expression were associated with poor prognosis, and the 5-year survival rate of patients with low ZNF521 expression was significantly higher than that of patients with high ZNF521 expression (Figure 2(a)). Kaplan–Meier curves and univariate analysis (log-rank) were further used to analyze the correlation between ZNF521 expression and patient prognosis according to TNM stage. In stage III gastric cancer, 5-year survival was significantly lower in patients with high ZNF521 expression compared with patients with low expression (*P* < 0.05; Figure 2(c)). For stage I, II, and IV disease, ZNF521 expression was not correlated with 5-year survival (*P* > 0.05; Figures 2(b) and 2(d)).

The prognostic value of ZNF521 on the basis of Affymetrix microarray data in gastric cancer was evaluated using the Kaplan–Meier Plotter database. High ZNF521 expression was associated with poor prognosis in gastric cancer (overall survival [OS]: HR = 1.79, 95% CI = 1.43–2.24, *P* = 3.1 × 10^−7^; progression-free survival (PFS): HR = 1.72, 95% CI = 1.35–2.2, *P* = 1.2 × 10^−5^; post-progression survival (PPS): HR = 1.94, 95% CI = 1.48–2.56, *P* = 1.4 × 10^−6^; Figures 2(e)–2(g)). The results of analysis of these databases showed that increased ZNF521 expression had a poor prognostic value in gastric cancer.

### 3.4. Effect of ZNF521 Overexpression on the Prognosis of Gastric Cancer Patients with Lymphatic Metastasis

We used the Kaplan–Meier Plotter database to investigate the relationship between ZNF521 expression and the clinical characteristics of gastric cancer patients. The results showed that higher expression of ZNF521 was associated with sex, stage, and Lauren classification, but not with differentiation (*P* < 0.05). Specifically, higher ZNF521 expression was correlated with worse OS in stage II (HR = 2.18, 95% CI = 1.13–4.19, *P* = 0.017) and stage IV (HR = 1.85, 95% CI = 1.24–2.75, *P* = 0.0022) patients, with PFS in stage II (HR = 1.99, 95% CI = 1.06–3.73, *P* = 0.029) and stage III (HR = 1.63, 95% CI = 1.12–2.39, *P* = 0.011) patients, with PPS in stage II, III, and IV patients (HR = 3.33, 95% CI = 1.65–6.71, *P* = 0.00037; HR = 1.6, 95% CI = 1.02–2.5, *P* = 0.037; and HR = 1.84, 95% CI = 1.17–2.91, *P* = 0.0076, respectively), but was not associated with OS, PFS, or PPS in stage I patients or OS and PFS in stage N0 patients. In particular, we analyzed the prognostic value of ZNF521 expression in gastric cancer patients with lymphatic metastasis. Higher ZNF521 expression was correlated with worse OS in stage N1 (HR = 2.28, 95% CI = 1.46–3.55, *P* = 2.00 × 10^−4^) and stage N3 (HR = 2.05, 95% CI = 1.16–3.63, *P* = 0.012) patients, worse PFS in stage N1, N2, and N3 patients (HR = 2, 95% CI = 1.32–3.03, *P* = 0.00082; HR = 1.6, 95% CI = 1.03–2.48, *P* = 0.036; and HR = 1.8, 95% CI = 1.01–3.19, *P* = 0.046, respectively), worse PPS in stage N0, N1, and N2 patients (HR = 4.09, 95% CI = 1.25–13.37, *P* = 0.012; HR = 1.98, 95% CI = 1.47–2.66, *P* = 4.40 × 10^−6^; and HR = 2.68, 95% CI = 1.64–4.37, *P* = 4.10 × 10^−5^, respectively; Table 2). Thus, ZNF521 expression may affect the prognosis of gastric cancer patients by promoting lymph node metastasis.

### 3.5. Relationship between ZNF521 Expression and Tumor Immune Infiltration

We next analyzed the relationship between ZNF521 expression and immune infiltration levels in 39 tumor types using the TIMER database (Figure S1). The results showed that ZNF521 expression was significantly correlated with tumor purity in 25 cancer types, B-cell infiltration levels in 23 cancer types, CD4+ T cells in 28 cancer types, CD4+ T cells in 21 cancer types, DCs in 23 cancer types, macrophages in 31 cancer types, and neutrophils in 25 cancer types (Table S1).

**Figure 3:**
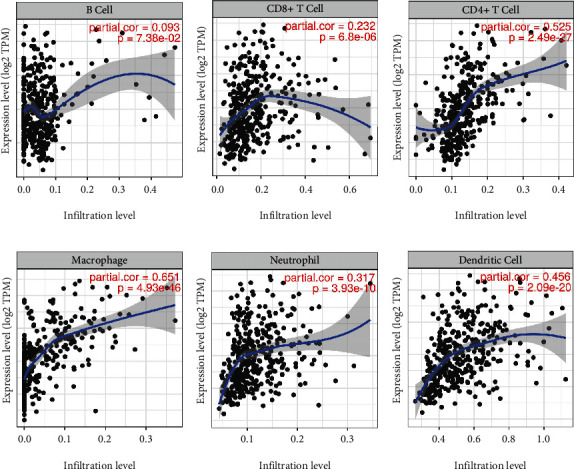
ZNF521 expression is correlated with the level of immune infiltration in STAD. (a) The expression of ZNF521 has no significant correlations with B Cells. (b)–(f) The expression of ZNF521 has positive correlations with infiltrating levels of CD8+ T cells, CD4+ T cells, macrophages, neutrophils, and dendritic cells.

Interestingly, we found that ZNF521 expression was associated with higher immune infiltration in gastric cancer and poor prognosis. ZNF521 expression was positively correlated with B-cell infiltration (*r* = 0.093, *P* = 0.074), CD8+ T cells (*r* = 0.232, *P* = 6.80 × 10^−6^), CD4+ T cells (*r* = 0.525, *P* = 2.49 × 10^−27^), macrophages (*r* = 0.651, *P* = 4.93 × 10^−46^), neutrophils (*r* = 0.317, *P* = 3.93 × 10^−10^), and DCs (*r* = 0.456, *P* = 2.09 × 10^−20^) in gastric cancer (Figure 3). These findings strongly suggest that ZNF521 plays a specific role in immune infiltration in gastric cancer.

### 3.6. Correlation Analysis between ZNF521 Expression and Immune Marker Sets

To further investigate the relationship between ZNF521 expression and the infiltration of different immune cell types, we analyzed the correlation between ZNF521 expression and markers of different immune cells, including CD8+ T cells, total T cells, B cells, monocytes, tumor-associated macrophages (TAMs), M1 and M2 macrophages, neutrophils, NK cells, DCs, Th1 cells, Th2 cells, Tfh cells, Th17 cells, regulatory T cells (Tregs), and exhausted T cells (Tex) in gastric cancer (Table 3). The results showed that ZNF521 expression was significantly related to most of the markers for various immune cells and T cells in gastric cancer.

**Figure 4:**
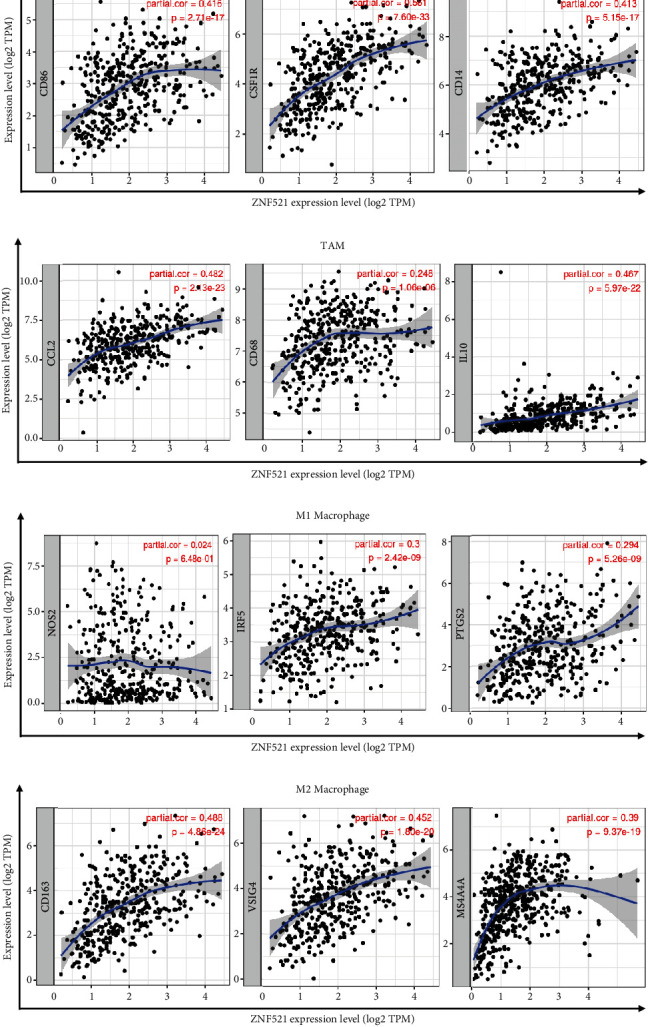
The correlation analysis between ZNF521 expression and immunological marker gene in STAD. Scatterplots of correlations between ZNF521 expression and gene markers of monocytes (a), TAMs (b), and M1 (c) and M2 macrophages (d) in STAD.

ZNF521 expression also had a strong correlation with markers of DCs, such as HLA-DPB1, HLA-DQB1, HLA-DRA, HLA-DPA1, BDCA-1 (CD1C), BDCA-4 (NRP1), and CD11c (ITGAX; Table 3). ZNF521 expression was also significantly correlated with marker genes of Tex, such as PD-1 (PDCD1), CTLA4, LAG3, TIM-3 (HAVCR2), and GZMB (Table 3). We also found that most markers of monocytes, TAMs, and M2 macrophages were significantly correlated with ZNF521 expression, but not with the expression of M1 marker genes (Table 3 and Figure 4). We further analyzed the correlation between ZNF521 expression and markers of monocytes, TAMs, and M1 and M2 macrophages in the GEPIA database of gastric cancer (Table S2). These results suggested that ZNF521 may regulate the polarization of macrophages in gastric cancer.

## 4. Discussion

The tumor microenvironment plays an important role in the dynamic regulation of cancer progression. Therapeutic strategies targeting the tumor microenvironment have emerged as promising cancer treatments. Immunotherapy has been approved in clinical trials and has broad application prospects [24]. Broadly, recent findings, including those presented herein, support the conclusion that immune cells including T and B lymphocytes, TAMs, DCs, natural killer cells, neutrophils, and myeloid suppressor cells playing an important role in the outcomes of gastric cancer patients.

A comprehensive analysis of tumor-infiltrating immune cells will help elucidate the mechanisms of tumor immune escape and provide opportunities for the development of new therapeutic strategies. ZNF521 is a multifunctional transcription cofactor that regulates many biological processes, such as hematopoietic differentiation, cell proliferation, autophagy, inhibiting EBF1, and promoting the development of B-cell leukemia [11, 25–28]. In this study, we found that ZNF521 is a valuable prognostic biomarker that is significantly correlated with cancer immune infiltration. Through database analysis, we showed that high ZNF521 expression was significantly associated with poor survival outcomes in ovarian cancer, gastric cancer, colon adenocarcinoma, bladder cancer, lung squamous cell carcinoma, and thyroid cancer. Specifically, in gastric cancer, higher ZNF521 expression was correlated with worse OS in stage II and stage IV patients, PFS in stage II and stage III patients, PPS in stage II, III, and IV patients, but was not associated with OS, PFS, or PPS in stage I patients or OS and PFS in stage N0 patients.

ZNF521 expression was correlated with levels of infiltrating CD4+ and CD8+ T cells, macrophages, neutrophils, and DCs in bladder cancer, lung squamous cell carcinoma, and gastric cancer, and was also correlated with diverse immune markers. Additionally, the correlation between ZNF521 expression and immune cell markers indicates that ZNF521 regulates tumor immunity in bladder cancer, lung squamous cell carcinoma, and gastric cancer. First, markers of M1 macrophages, such as inducible nitric oxide synthase, interferon regulatory factor 5 (IRF5), and prostaglandin-endoperoxide synthase 2 (PTGS2) were not correlated or were only weakly correlated with ZNF521 expression, while markers of M2 macrophages, such as CD163, V-set immunoglobulin-domain-containing 4 (VSIG4), and MS4A4A were strongly correlated with ZNF521 expression (Table 2). These results reveal the potential regulatory role of ZNF521 in the polarization of TAMs.

DCs are the most powerful full-time antigen-presenting cells of the immune system and play a key role in the initiation and regulation of immune responses [29]. In most cases, vaccines against cancer antigens rely on DCs, which can be used to promote individualized treatment via anti-tumor immunity [30, 31]. ZNF521 is strongly related to markers of DCs including HLA-DPB1, HLA-DQB1, HLA-DRA, HLA-DPA1, BDCA-1 (CD1C), BDCA-4 (NRP1), and CD11c (ITGAX).

Together, these findings suggest that ZNF521 is a valuable prognostic biomarker for gastric and other cancers that is significantly correlated with immune infiltration.

## Figures and Tables

**Table 1:** Relationship of ZNF521 expression with pathological characteristics of gastric cancer.

Clinical parameters	ZNF521			
	Low	High	*t*/*χ*^2^	*P*-value
Age (years)	58.21 ± 11.21	59.64 ± 12.71	−1.205	0.229
**Gender**			0.894	0.201
Male	124 (39.9%)	187 (60.1%)		
Female	56 (44.8%)	69 (55.2%)		
**Location**			2.819	**<0.01**
Proximal	17 (30.9%)	38 (69.1%)		
Middle	69 (42.3%)	94 (57.7%)		
Distal	94 (43.1%)	124 (56.9%)		
**Size**			6.917	**0.006**
<5 cm	119 (46.5%)	137 (53.5%)		
≥5 cm	61 (33.9%)	119 (66.1%)		
**Lauren classification**			9.616	**0.001**
Intestinal	108 (48.4%)	115 (51.6%)		
Diffuse	72 (33.8%)	141 (66.2%)		
**Histology classification**			4.326	**<0.01**
Papillary adenocarcinoma	4 (25.0%)	12 (75.0%)		
Tubular adenocarcinoma	131 (40.2%)	195 (59.8%)		
Mucinous adenocarcinoma	12 (41.4%)	17 (58.6%)		
Signet-ring cell carcinoma	33 (50.8%)	32 (49.2%)		
**Histologic differentiation**			1.572	**<0.01**
Well	6 (46.2%)	7 (53.8%)		
Moderately	52 (40.6%)	76 (59.4%)		
Poorly	122 (41.6%)	171 (58.4%)		
Others	0 (0.0%)	2 (100.0%)		
**Invasion depth**			1.486	**<0.01**
T1	27 (47.4%)	30 (52.6%)		
T2	47 (43.1%)	62 (56.9%)		
T3	96 (39.3%)	148 (60.7%)		
T4	10 (38.5%)	16 (61.5%)		
**Lymphatic metastasis**			1.200	0.160
No	74 (44.6%)	92 (55.4%)		
Yes	106 (39.3%)	164 (60.7%)		
**Regional lymph nodes**			4.297	**<0.01**
PN0	74 (44.6%)	92 (55.4%)		
PN1	57 (41.9%)	79 (58.1%)		
PN2	40 (40.4%)	59 (59.6%)		
PN3	9 (25.7%)	26 (74.3%)		
**Distant metastasis**			**6.631**	**0.007**
No	164 (43.7%)	211 (56.3%)		
Yes	16 (26.2%)	45 (73.8%)		
**TNM stages**			**6.783**	**<0.01**
I	42 (46.7%)	48 (53.3%)		
II	45 (43.3%)	59 (56.7%)		
III	74 (42.8%)	99 (57.2%)		
IV	19 (27.5%)	50 (72.5%)		

Bold values indicate that statistical significance was set at *P* < 0.05.

**Table 2:** The correlation of ZNF521 mRNA expression and clinical prognosis in gastric cancer with different clinicopathological factors by Kaplan–Meier plotter.

Clinicopathological characteristics	*N*	OS	*P*-value	*N*	FP	*P*-value	*N*	PPS	*P*-value
		*n* = 881			*n* = 645			*n* = 503	
		HR			HR			HR	
Gender									
Female	236	2.41 (1.47–3.96)	**0.00031**	201	2.24 (1.38–3.63)	**0.0008**	149	2.61 (1.49–4.59)	**0.00053**
Male	544	1.71 (1.27–2.31)	**0.00036**	437	1.67 (1.24–2.23)	**0.00056**	348	1.86 (1.32–2.63)	**3.00 × 10** ^**−4**^
Stage									
1	67	1.76 (0.57–5.42)	0.32	60	1.68 (0.54–5.2)	0.36	31		
2	140	2.18 (1.13–4.19)	**0.017**	131	1.99 (1.06–3.73)	**0.029**	105	3.33 (1.65–6.71)	**0.00037**
3	205	1.44 (0.98–2.13)	0.064	186	1.63 (1.12–2.39)	**0.011**	142	1.6 (1.02–2.5)	**0.037**
4	148	1.85 (1.24–2.75)	**0.0022**	141	1.44 (0.98–2.11)	0.064	104	1.84 (1.17–2.91)	**0.0076**
Stage T									
1	14			14			3		
2	241	1.9 (1.24–2.9)	**0.0025**	239	1.71 (1.13–2.58)	**0.0099**	196	2.29 (1.46–3.59)	**0.00019**
3	204	1.22 (0.83–1.78)	0.37	204	0.81 (0.56–1.18)	0.27	150	1.27 (0.85–1.89)	0.25
4	38	3.38 (1.37–8.32)	**0.0051**	39	2.7 (1.19–6.14)	**0.014**	29		
Stage N									
0	74	2.15 (0.93–4.98)	0.068	72	2.05 (0.89–4.74)	0.086	41	4.09 (1.25–13.37)	**0.012**
1	225	2.28 (1.46–3.55)	**2.00 × 10** ^**−4**^	222	2 (1.32–3.03)	**0.00082**	169	1.98 (1.47–2.66)	**4.40 × 10** ^**−6**^
2	121	1.53 (0.95–2.47)	0.08	125	1.6 (1.03–2.48)	**0.036**	105	2.68 (1.64–4.37)	**4.10 × 10** ^**−5**^
3	76	2.05 (1.16–3.63)	**0.012**	76	1.8 (1.01–3.19)	**0.046**	63	1.55 (0.94–2.53)	0.081
1 + 2 + 3	422	1.89 (1.43–2.49)	**4.30 × 10** ^**−6**^	423	1.77 (1.36–2.3)	**1.40 × 10** ^**−5**^	437	1.91 (1.07–3.42)	**0.026**
Stage M									
0	444	1.84 (1.38–2.46)	**2.40 × 10** ^**−5**^	443	1.77 (1.34–2.32)	**3.80 × 10** ^**−5**^	342	2.03 (1.5–2.74)	**2.50 × 10** ^**−6**^
1	56	1.9 (1.05–3.44)	**0.031**	56	1.54 (0.85–2.79)	0.15	36	2.65 (1–7.06)	**0.043**
Lauren classification									
Intestinal	320	1.74 (1.21–2.5)	**0.0024**	263	1.7 (1.2–2.42)	**0.0026**	192	1.75 (1.16–2.63)	**0.007**
Diffuse	241	1.71 (1.15–2.54)	**0.0079**	231	1.86 (1.24–2.8)	**0.0024**	176	1.89 (1.29–2.77)	**0.00095**
Mixed	32	3.32 (1.09–10.12)	**0.026**	28	1.9(0.54–6.73)	0.31	16		
Differentiation									
Poor	165	1.22 (0.75–1.97)	0.42	121	1.23 (0.73–2.05)	0.44	49	1.31 (0.69–2.48)	0.41
Moderate	67	1.55 (0.79–3.03)	0.19	67	1.9 (0.99–3.65)	0.05	24	0.42 (0.16–1.16)	0.086
Well	32			5			0		

Bold values indicate that statistical significance was set at *P* < 0.05.

**Table 3:** The correlation analysis between ZNF521 and relate genes and markers of immune cells in TIMER.

Description	Gene markers	STAD		Description	Gene markers	STAD	
		purity				purity	
		cor	*P*-value			cor	*P*-value
**CD8+ T cells**	CD8A	−0.219772	∗∗∗	**Dendritic cells**	HLA-DPB1	−0.29309	∗∗∗
	CD8B	−0.121084	∗		HLA-DQB1	−0.28247	∗∗∗
**T cells (general)**	CD3D	−0.315001	∗∗∗		HLA-DRA	−0.276107	∗∗∗
	CD3E	−0.334622	∗∗∗		HLA-DPA1	−0.276393	∗∗∗
	CD2	−0.302668	∗∗∗		BDCA-1 (CD1C)	−0.284624	∗∗∗
**B cells**	CD19	−0.218492	∗∗∗		BDCA-4 (NRP1)	−0.172732	∗∗∗
	CD79A	−0.268382	∗∗∗		CD11c (ITGAX)	−0.224098	∗∗∗
**Monocytes**	CD86	−0.285611	∗∗∗	**Th1**	T -bet (TBX21)	−0.253967	∗∗∗
	CD115 (CSF1R)	−0.208257	∗∗∗		STAT4	−0.245164	∗∗∗
**TAM**	CCL2	−0.204723	∗∗∗		STAT1	−0.104384	0.0419837
	CD68	−0.159045	∗		IFN-*γ* (IFNG)	−0.189716	∗∗
	IL10	−0.25374	∗∗∗		TNF-*α* (TNF)	−0.280664	∗∗∗
**M1 macrophage**	INOS (NOS2)	−0.094312	0.066282	**Th2**	GATA3	−0.174418	∗∗
	IRF5	−0.111113	0.0303446		STAT6	0.0106501	0.8360634
	COX2(PTGS2)	−0.125946	0.0140167		STAT5A	−0.131781	0.0101217
**M2 macrophage**	CD163	−0.190151	∗∗∗		IL13	−0.001852	0.9712901
	VSIG4	−0.16597	∗	**Tfh**	BCL6	−0.134597	∗
	MS4A4A	−0.190671	∗∗∗		IL21	−0.13569	∗
**Neutrophils**	CD66b (CEACAM8)	0.0206156	0.688722	**Th17**	STAT3	−0.07143	0.1646457
	CD11b (ITGAM)	−0.16399	∗		IL17A	−0.122095	0.0172588
	CCR7	−0.291648	∗∗∗	**Treg**	FOXP3	−0.241316	∗∗∗
**Natural killer cells**	KIR2DL1	−0.076515	0.1365359		CCR8	−0.167921	∗
	KIR2DL3	−0.131514	0.0102763		STAT5B	−0.022563	0.6610653
	KIR2DL4	−0.164984	∗		TGF*β* (TGFB1)	−0.168879	∗∗
	KIR3DL1	−0.124038	0.0155496	**T cell exhaustion**	PD-1 (PDCD1)	−0.174787	∗∗
	KIR3DL2	−0.161244	∗		CTLA4	−0.197054	∗∗
	KIR3DL3	−0.019629	0.7028965		LAG3	−0.227405	∗∗∗
	KIR2DS4	−0.121695	0.0176306		TIM-3 (HAVCR2)	−0.244911	∗∗∗
					GZMB	−0.253689	∗∗∗

∗, ∗∗, and ∗∗∗ indicate that statistical significance was set at *P* < 0.05.

## Data Availability

The data used to support the findings of this study are included within the article.

## Supplementary Materials

Supplementary Materials Figure S1. Correlation analysis between ZNF521 expression and the level of immune infiltration in 39 tumor types using the TIMER database.

Supplementary Materials Table S1. The correlations of ZNF521 expression with immune infiltration levels in 39 cancer types from TIMER. Table S2. Correlation analysis between ZNF521 and relate genes and markers of monocyte and macrophages in GEPIA. ∗*P* < 0.01; ∗∗*P* < 0.001; ∗∗∗*P* < 0.0001.
