# Supplementary figures and images for: ZNF521 Is Correlated with Tumor Immune Cell Infiltration and Act as a Valuable Prognostic Biomarker in Gastric Cancer
Source: Gastroenterol Res Pract. 2022 Oct 19;2022:5288075. doi: 10.1155/2022/5288075 (PMC9606838; doi:10.1155/2022/5288075)

## Slide 1
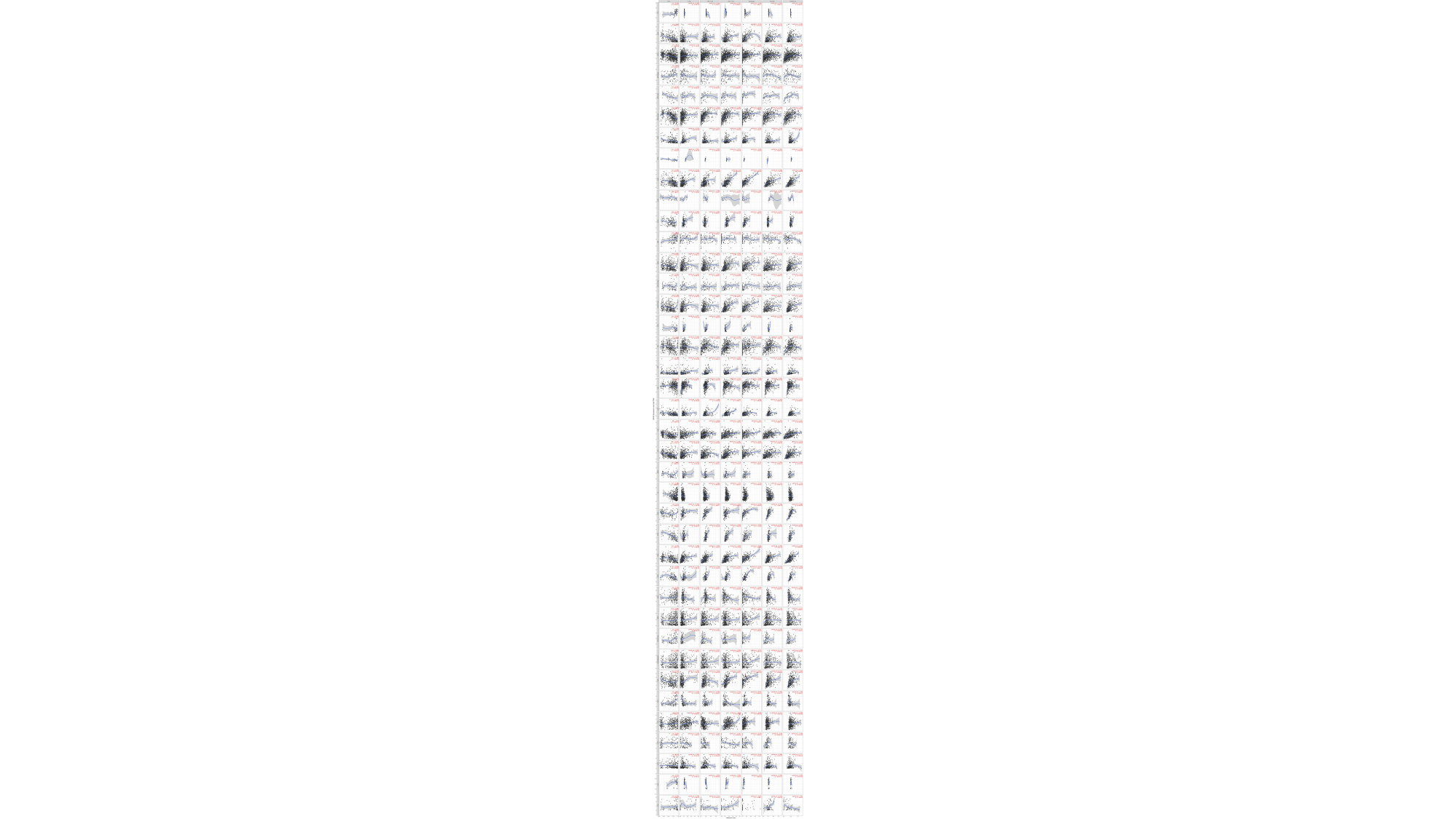

Supplement: Supplementary Materials — Figure S1. Correlation analysis between ZNF521 expression and the level of immune infiltration in 39 tumor types using the TIMER database. [file 5288075.f1.pptx]
