# Supplementary material for: ZNF521 Is Correlated with Tumor Immune Cell Infiltration and Act as a Valuable Prognostic Biomarker in Gastric Cancer
Source: Gastroenterol Res Pract. 2022 Oct 19;2022:5288075. doi: 10.1155/2022/5288075 (PMC9606838; doi:10.1155/2022/5288075)
Supplement: Supplementary Materials — Table S1. The correlations of ZNF521 expression with immune infiltration levels in 39 cancer types from TIMER. Table S2. Correlation analysis between ZNF521 and relate genes and markers of monocyte and macrophages in GEPIA. ∗P < 0.01; ∗∗P < 0.001; ∗∗∗P < 0.0001. [file 5288075.f2.docx]

Table S1. The correlations of ZNF521 expression with immune infiltration levels in 39 cancer types from TIMER.

| cancer | variable | partial.cor | p |
| --- | --- | --- | --- |
| ACC | Purity | 0.451283 | 5.45E-05 |
| ACC | B Cell | 0.382043 | 0.000852 |
| ACC | CD8+ T Cell | 0.003298 | 0.977907 |
| ACC | CD4+ T Cell | 0.121353 | 0.306434 |
| ACC | Macrophage | 0.163857 | 0.165987 |
| ACC | Neutrophil | -0.03331 | 0.779675 |
| ACC | Dendritic Cell | 0.080919 | 0.49615 |
| BLCA | Purity | -0.46047 | 9.15E-21 |
| BLCA | B Cell | -0.01878 | 0.721439 |
| BLCA | CD8+ T Cell | 0.135135 | 0.009644 |
| BLCA | CD4+ T Cell | 0.174295 | 0.000825 |
| BLCA | Macrophage | 0.515889 | 3.27E-26 |
| BLCA | Neutrophil | 0.151427 | 0.00383 |
| BLCA | Dendritic Cell | 0.084514 | 0.106967 |
| BRCA | Purity | -0.32996 | 1.06E-26 |
| BRCA | B Cell | 0.050128 | 0.117385 |
| BRCA | CD8+ T Cell | 0.352281 | 6.80E-30 |
| BRCA | CD4+ T Cell | 0.313378 | 2.30E-23 |
| BRCA | Macrophage | 0.32384 | 1.96E-25 |
| BRCA | Neutrophil | 0.274768 | 6.20E-18 |
| BRCA | Dendritic Cell | 0.269113 | 3.06E-17 |
| BRCA-Basal | Purity | 0.199472 | 0.02343 |
| BRCA-Basal | B Cell | 0.120413 | 0.182814 |
| BRCA-Basal | CD8+ T Cell | 0.13014 | 0.151374 |
| BRCA-Basal | CD4+ T Cell | 0.207817 | 0.021623 |
| BRCA-Basal | Macrophage | 0.123742 | 0.165731 |
| BRCA-Basal | Neutrophil | 0.041979 | 0.663239 |
| BRCA-Basal | Dendritic Cell | 0.119149 | 0.206717 |
| BRCA-Her2 | Purity | -0.4468 | 0.000389 |
| BRCA-Her2 | B Cell | -0.10612 | 0.427855 |
| BRCA-Her2 | CD8+ T Cell | 0.061414 | 0.649959 |
| BRCA-Her2 | CD4+ T Cell | 0.055371 | 0.679733 |
| BRCA-Her2 | Macrophage | 0.57304 | 2.59E-06 |
| BRCA-Her2 | Neutrophil | 0.073497 | 0.58349 |
| BRCA-Her2 | Dendritic Cell | 0.197406 | 0.14475 |
| BRCA-Luminal | Purity | -0.49309 | 8.53E-35 |
| BRCA-Luminal | B Cell | 0.071324 | 0.097475 |
| BRCA-Luminal | CD8+ T Cell | 0.435514 | 2.90E-26 |
| BRCA-Luminal | CD4+ T Cell | 0.383255 | 3.38E-20 |
| BRCA-Luminal | Macrophage | 0.433485 | 3.08E-26 |
| BRCA-Luminal | Neutrophil | 0.328503 | 6.67E-15 |
| BRCA-Luminal | Dendritic Cell | 0.32231 | 2.13E-14 |
| CESC | Purity | -0.25961 | 1.16E-05 |
| CESC | B Cell | 0.317881 | 6.39E-08 |
| CESC | CD8+ T Cell | 0.01175 | 0.846475 |
| CESC | CD4+ T Cell | 0.187944 | 0.001678 |
| CESC | Macrophage | 0.303113 | 2.70E-07 |
| CESC | Neutrophil | -0.01954 | 0.746062 |
| CESC | Dendritic Cell | 0.090685 | 0.132883 |
| CHOL | Purity | -0.42327 | 0.010105 |
| CHOL | B Cell | 0.690986 | 4.33E-06 |
| CHOL | CD8+ T Cell | 0.582649 | 0.00024 |
| CHOL | CD4+ T Cell | 0.514943 | 0.00155 |
| CHOL | Macrophage | 0.606186 | 0.000114 |
| CHOL | Neutrophil | 0.562072 | 0.000442 |
| CHOL | Dendritic Cell | 0.569142 | 0.00036 |
| COAD | Purity | -0.36646 | 2.21E-14 |
| COAD | B Cell | 0.163306 | 0.000986 |
| COAD | CD8+ T Cell | 0.278135 | 1.20E-08 |
| COAD | CD4+ T Cell | 0.600272 | 1.03E-40 |
| COAD | Macrophage | 0.679333 | 5.44E-56 |
| COAD | Neutrophil | 0.525702 | 7.11E-30 |
| COAD | Dendritic Cell | 0.565672 | 2.24E-35 |
| DLBC | Purity | -0.24707 | 0.114691 |
| DLBC | B Cell | 0.507439 | 0.031587 |
| DLBC | CD8+ T Cell | -0.083 | 0.720586 |
| DLBC | CD4+ T Cell | -0.04686 | 0.840167 |
| DLBC | Macrophage | -0.01962 | 0.932716 |
| DLBC | Neutrophil | 0.365887 | 0.102854 |
| DLBC | Dendritic Cell | 0.224573 | 0.327736 |
| ESCA | Purity | -0.22863 | 0.001964 |
| ESCA | B Cell | 0.203318 | 0.006339 |
| ESCA | CD8+ T Cell | 0.082758 | 0.269387 |
| ESCA | CD4+ T Cell | 0.151709 | 0.042636 |
| ESCA | Macrophage | 0.563259 | 1.86E-16 |
| ESCA | Neutrophil | 0.07659 | 0.306828 |
| ESCA | Dendritic Cell | 0.081552 | 0.276452 |
| GBM | Purity | 0.331813 | 7.02E-05 |
| GBM | B Cell | 0.071528 | 0.426076 |
| GBM | CD8+ T Cell | 0.01534 | 0.862477 |
| GBM | CD4+ T Cell | 0.159288 | 0.0681 |
| GBM | Macrophage | 0.117992 | 0.17952 |
| GBM | Neutrophil | -0.07684 | 0.384869 |
| GBM | Dendritic Cell | -0.00168 | 0.984743 |
| HNSC | Purity | -0.20117 | 6.75E-06 |
| HNSC | B Cell | 0.29522 | 5.00E-11 |
| HNSC | CD8+ T Cell | 0.203265 | 8.18E-06 |
| HNSC | CD4+ T Cell | 0.492327 | 1.14E-30 |
| HNSC | Macrophage | 0.522009 | 4.10E-35 |
| HNSC | Neutrophil | 0.213674 | 2.37E-06 |
| HNSC | Dendritic Cell | 0.417592 | 9.18E-22 |
| HNSC-HPVpos | Purity | -0.17245 | 0.104089 |
| HNSC-HPVpos | B Cell | 0.022235 | 0.845783 |
| HNSC-HPVpos | CD8+ T Cell | -0.05509 | 0.631891 |
| HNSC-HPVpos | CD4+ T Cell | 0.362929 | 0.000805 |
| HNSC-HPVpos | Macrophage | 0.374656 | 0.000379 |
| HNSC-HPVpos | Neutrophil | 0.083965 | 0.444864 |
| HNSC-HPVpos | Dendritic Cell | 0.214309 | 0.051714 |
| HNSC-HPVneg | Purity | -0.20835 | 2.61E-05 |
| HNSC-HPVneg | B Cell | 0.368174 | 4.30E-14 |
| HNSC-HPVneg | CD8+ T Cell | 0.258107 | 2.12E-07 |
| HNSC-HPVneg | CD4+ T Cell | 0.537188 | 6.54E-31 |
| HNSC-HPVneg | Macrophage | 0.56206 | 3.43E-34 |
| HNSC-HPVneg | Neutrophil | 0.251613 | 4.64E-07 |
| HNSC-HPVneg | Dendritic Cell | 0.47224 | 2.18E-23 |
| KICH | Purity | -0.0417 | 0.739568 |
| KICH | B Cell | 0.277375 | 0.025289 |
| KICH | CD8+ T Cell | 0.396021 | 0.001093 |
| KICH | CD4+ T Cell | -0.03647 | 0.773023 |
| KICH | Macrophage | 0.541429 | 3.21E-06 |
| KICH | Neutrophil | -0.15181 | 0.227355 |
| KICH | Dendritic Cell | 0.267297 | 0.031355 |
| KIRC | Purity | -0.18989 | 3.99E-05 |
| KIRC | B Cell | 0.02788 | 0.551315 |
| KIRC | CD8+ T Cell | 0.251665 | 9.39E-08 |
| KIRC | CD4+ T Cell | 0.391862 | 2.47E-18 |
| KIRC | Macrophage | 0.322992 | 2.45E-12 |
| KIRC | Neutrophil | 0.294038 | 1.38E-10 |
| KIRC | Dendritic Cell | 0.180253 | 0.000111 |
| KIRP | Purity | -0.19766 | 0.001387 |
| KIRP | B Cell | 0.327096 | 8.51E-08 |
| KIRP | CD8+ T Cell | 0.414455 | 3.94E-12 |
| KIRP | CD4+ T Cell | 0.233016 | 0.000159 |
| KIRP | Macrophage | 0.110764 | 0.08109 |
| KIRP | Neutrophil | 0.263485 | 1.81E-05 |
| KIRP | Dendritic Cell | 0.425706 | 1.08E-12 |
| LGG | Purity | -0.08046 | 0.078553 |
| LGG | B Cell | 0.290579 | 9.38E-11 |
| LGG | CD8+ T Cell | 0.191918 | 2.40E-05 |
| LGG | CD4+ T Cell | 0.146635 | 0.001336 |
| LGG | Macrophage | 0.258756 | 1.13E-08 |
| LGG | Neutrophil | 0.208959 | 4.37E-06 |
| LGG | Dendritic Cell | 0.172158 | 0.00016 |
| LIHC | Purity | -0.40331 | 5.76E-15 |
| LIHC | B Cell | 0.093976 | 0.081771 |
| LIHC | CD8+ T Cell | 0.231627 | 1.51E-05 |
| LIHC | CD4+ T Cell | 0.352075 | 1.78E-11 |
| LIHC | Macrophage | 0.286409 | 7.34E-08 |
| LIHC | Neutrophil | 0.306315 | 6.28E-09 |
| LIHC | Dendritic Cell | 0.2772 | 2.05E-07 |
| LUAD | Purity | -0.3599 | 1.49E-16 |
| LUAD | B Cell | 0.144242 | 0.001464 |
| LUAD | CD8+ T Cell | 0.209193 | 3.22E-06 |
| LUAD | CD4+ T Cell | 0.32893 | 1.12E-13 |
| LUAD | Macrophage | 0.340416 | 1.27E-14 |
| LUAD | Neutrophil | 0.354957 | 8.68E-16 |
| LUAD | Dendritic Cell | 0.401349 | 2.60E-20 |
| LUSC | Purity | -0.3411 | 1.73E-14 |
| LUSC | B Cell | 0.270004 | 2.50E-09 |
| LUSC | CD8+ T Cell | 0.251174 | 2.87E-08 |
| LUSC | CD4+ T Cell | 0.46062 | 2.53E-26 |
| LUSC | Macrophage | 0.431168 | 5.13E-23 |
| LUSC | Neutrophil | 0.433604 | 3.06E-23 |
| LUSC | Dendritic Cell | 0.512413 | 4.32E-33 |
| MESO | Purity | -0.15203 | 0.162289 |
| MESO | B Cell | 0.217626 | 0.046746 |
| MESO | CD8+ T Cell | -0.06734 | 0.54279 |
| MESO | CD4+ T Cell | 0.180144 | 0.101057 |
| MESO | Macrophage | 0.16081 | 0.143935 |
| MESO | Neutrophil | -0.08355 | 0.449872 |
| MESO | Dendritic Cell | 0.065942 | 0.551198 |
| OV | Purity | -0.08569 | 0.059583 |
| OV | B Cell | -0.17732 | 9.39E-05 |
| OV | CD8+ T Cell | -0.08609 | 0.059454 |
| OV | CD4+ T Cell | -0.0701 | 0.125083 |
| OV | Macrophage | 0.10272 | 0.024412 |
| OV | Neutrophil | -0.13141 | 0.003927 |
| OV | Dendritic Cell | -0.12971 | 0.004422 |
| PAAD | Purity | -0.21019 | 0.005649 |
| PAAD | B Cell | 0.361837 | 1.16E-06 |
| PAAD | CD8+ T Cell | 0.592212 | 1.45E-17 |
| PAAD | CD4+ T Cell | 0.275689 | 0.000286 |
| PAAD | Macrophage | 0.75823 | 3.25E-33 |
| PAAD | Neutrophil | 0.622468 | 1.00E-19 |
| PAAD | Dendritic Cell | 0.68265 | 8.45E-25 |
| PCPG | Purity | -0.49661 | 7.58E-12 |
| PCPG | B Cell | 0.079694 | 0.305947 |
| PCPG | CD8+ T Cell | 0.316435 | 3.10E-05 |
| PCPG | CD4+ T Cell | 0.328157 | 1.50E-05 |
| PCPG | Macrophage | 0.295434 | 0.000111 |
| PCPG | Neutrophil | 0.306739 | 5.54E-05 |
| PCPG | Dendritic Cell | 0.295643 | 0.000105 |
| PRAD | Purity | -0.33391 | 2.56E-12 |
| PRAD | B Cell | 0.486239 | 7.75E-26 |
| PRAD | CD8+ T Cell | 0.462135 | 2.12E-23 |
| PRAD | CD4+ T Cell | 0.449287 | 8.21E-22 |
| PRAD | Macrophage | 0.61626 | 7.23E-45 |
| PRAD | Neutrophil | 0.464394 | 1.55E-23 |
| PRAD | Dendritic Cell | 0.592818 | 9.72E-41 |
| READ | Purity | -0.36816 | 7.63E-06 |
| READ | B Cell | 0.178991 | 0.035007 |
| READ | CD8+ T Cell | 0.148138 | 0.081796 |
| READ | CD4+ T Cell | 0.41916 | 2.81E-07 |
| READ | Macrophage | 0.516459 | 7.62E-11 |
| READ | Neutrophil | 0.313615 | 0.00018 |
| READ | Dendritic Cell | 0.475965 | 3.18E-09 |
| SARC | Purity | 0.106348 | 0.096752 |
| SARC | B Cell | -0.13219 | 0.041159 |
| SARC | CD8+ T Cell | -0.06056 | 0.350237 |
| SARC | CD4+ T Cell | -0.13153 | 0.042191 |
| SARC | Macrophage | -0.02524 | 0.700241 |
| SARC | Neutrophil | 0.041154 | 0.524021 |
| SARC | Dendritic Cell | -0.25221 | 7.52E-05 |
| SKCM | Purity | 0.055173 | 0.238627 |
| SKCM | B Cell | 0.135675 | 0.004015 |
| SKCM | CD8+ T Cell | 0.087815 | 0.066339 |
| SKCM | CD4+ T Cell | 0.087819 | 0.063883 |
| SKCM | Macrophage | 0.221847 | 1.86E-06 |
| SKCM | Neutrophil | 0.174835 | 0.000187 |
| SKCM | Dendritic Cell | 0.070767 | 0.135651 |
| SKCM-Primary | Purity | 0.28145 | 0.003977 |
| SKCM-Primary | B Cell | 0.215663 | 0.030314 |
| SKCM-Primary | CD8+ T Cell | 0.227485 | 0.022148 |
| SKCM-Primary | CD4+ T Cell | 0.137123 | 0.171506 |
| SKCM-Primary | Macrophage | 0.300677 | 0.002249 |
| SKCM-Primary | Neutrophil | 0.338626 | 0.000569 |
| SKCM-Primary | Dendritic Cell | 0.130785 | 0.192363 |
| SKCM-Metastasis | Purity | -0.00352 | 0.947239 |
| SKCM-Metastasis | B Cell | 0.081013 | 0.132596 |
| SKCM-Metastasis | CD8+ T Cell | 0.031331 | 0.567111 |
| SKCM-Metastasis | CD4+ T Cell | 0.056497 | 0.296072 |
| SKCM-Metastasis | Macrophage | 0.190667 | 0.000328 |
| SKCM-Metastasis | Neutrophil | 0.112637 | 0.034907 |
| SKCM-Metastasis | Dendritic Cell | 0.024711 | 0.647871 |
| STAD | Purity | -0.18924 | 0.000207 |
| STAD | B Cell | 0.09319 | 0.073785 |
| STAD | CD8+ T Cell | 0.231527 | 6.80E-06 |
| STAD | CD4+ T Cell | 0.525138 | 2.49E-27 |
| STAD | Macrophage | 0.651308 | 4.93E-46 |
| STAD | Neutrophil | 0.317468 | 3.93E-10 |
| STAD | Dendritic Cell | 0.455522 | 2.09E-20 |
| TGCT | Purity | 0.327264 | 4.91E-05 |
| TGCT | B Cell | -0.20501 | 0.012742 |
| TGCT | CD8+ T Cell | -0.05836 | 0.482611 |
| TGCT | CD4+ T Cell | 0.10314 | 0.215407 |
| TGCT | Macrophage | 0.356942 | 9.09E-06 |
| TGCT | Neutrophil | 0.096146 | 0.246684 |
| TGCT | Dendritic Cell | -0.07981 | 0.338249 |
| THCA | Purity | 0.01974 | 0.663247 |
| THCA | B Cell | 0.28167 | 3.06E-10 |
| THCA | CD8+ T Cell | -0.20349 | 6.00E-06 |
| THCA | CD4+ T Cell | 0.242105 | 6.12E-08 |
| THCA | Macrophage | 0.247823 | 2.90E-08 |
| THCA | Neutrophil | 0.121134 | 0.007385 |
| THCA | Dendritic Cell | 0.096018 | 0.034515 |
| THYM | Purity | 0.097198 | 0.299283 |
| THYM | B Cell | -0.1527 | 0.104807 |
| THYM | CD8+ T Cell | -0.12938 | 0.170088 |
| THYM | CD4+ T Cell | -0.18748 | 0.048799 |
| THYM | Macrophage | 0.125131 | 0.184664 |
| THYM | Neutrophil | 0.35974 | 8.46E-05 |
| THYM | Dendritic Cell | -0.15917 | 0.090743 |
| UCEC | Purity | -0.08003 | 0.171117 |
| UCEC | B Cell | 0.069219 | 0.240781 |
| UCEC | CD8+ T Cell | 0.262262 | 6.23E-06 |
| UCEC | CD4+ T Cell | 0.119779 | 0.041168 |
| UCEC | Macrophage | 0.183763 | 0.001613 |
| UCEC | Neutrophil | 0.089245 | 0.127474 |
| UCEC | Dendritic Cell | 0.171469 | 0.00329 |
| UCS | Purity | 0.072115 | 0.604312 |
| UCS | B Cell | 0.11728 | 0.402961 |
| UCS | CD8+ T Cell | -0.22903 | 0.099017 |
| UCS | CD4+ T Cell | 0.365441 | 0.007129 |
| UCS | Macrophage | 0.319756 | 0.019596 |
| UCS | Neutrophil | 0.024708 | 0.860595 |
| UCS | Dendritic Cell | 0.234695 | 0.090726 |
| UVM | Purity | 0.005747 | 0.960174 |
| UVM | B Cell | 0.188491 | 0.105336 |
| UVM | CD8+ T Cell | -0.12047 | 0.296666 |
| UVM | CD4+ T Cell | 0.269131 | 0.018728 |
| UVM | Macrophage | 0.206714 | 0.10124 |
| UVM | Neutrophil | 0.273249 | 0.016195 |
| UVM | Dendritic Cell | -0.37032 | 0.001163 |

Table S2. Correlation analysis between ZNF521 and relate genes and markers of monocyte and macrophages in GEPIA. *P < 0.01;**P < 0.001;***P < 0.0001.

| **Description** | **Gene markers** | **STAD** | | | |
| --- | --- | --- | --- | --- | --- |
|  |  | **Tumor** | | **Normal** | |
|  |  | R | P | R | P |
| **Monocyte** | CD86 | 0.42 | *** | 0.031 | 0.86 |
|  | CD115 | 0.59 | *** | 0.34 | 0.044 |
| **TAM** | CCL2 | 0.51 | *** | 0.46 | * |
|  | CD68 | 0.29 | *** | −0.22 | 0.19 |
|  | IL10 | 0.52 | *** | 0.27 | 0.11 |
| **M1 Macrophage** | NOS2 | 0.049 | 0.33 | 0.46 | * |
|  | IRF5 | 0.31 | *** | −0.35 | 0.035 |
|  | PTGS2 | 031 | *** | 0.7 | *** |
| **M2 Macrophage** | CD163 | 0.43 | *** | 0.64 | *** |
|  | VSIG4 | 0.46 | *** | 0.59 | ** |
|  | MS4A4A | 0.51 | *** | 0.7 | *** |
